# Patterns of cannabis use during adolescence and their association with harmful substance use behaviour: findings from a UK birth cohort

**DOI:** 10.1136/jech-2016-208503

**Published:** 2017-06-07

**Authors:** Michelle Taylor, Simon M Collin, Marcus R Munafò, John MacLeod, Matthew Hickman, Jon Heron

**Affiliations:** 1 School of Social and Community Medicine, University of Bristol, Bristol, UK; 2 MRC Integrative Epidemiology Unit (IEU), University of Bristol, Bristol, UK; 3 UK Centre for Tobacco and Alcohol Studies (UKCTAS), School of Experimental Psychology, University of Bristol, Bristol, UK; 4 Centre for Child and Adolescent Health, University of Bristol, Bristol, UK

**Keywords:** LONGITUDINAL STUDIES, DRUG MISUSE, ADOLESCENTS CG, Cohort studies

## Abstract

**Background:**

Evidence on the role of cannabis as a gateway drug is inconsistent. We characterise patterns of cannabis use among UK teenagers aged 13–18 years, and assess their influence on problematic substance use at age 21 years.

**Methods:**

We used longitudinal latent class analysis to derive trajectories of cannabis use from self-report measures in a UK birth cohort. We investigated (1) factors associated with latent class membership and (2) whether latent class membership predicted subsequent nicotine dependence, harmful alcohol use and recent use of other illicit drugs at age 21 years.

**Results:**

5315 adolescents had three or more measures of cannabis use from age 13 to 18 years. Cannabis use patterns were captured as four latent classes corresponding to ‘non-users’ (80.1%), ‘late-onset occasional’ (14.2%), ‘early-onset occasional’ (2.3%) and ‘regular’ users (3.4%). Sex, mother's substance use, and child's tobacco use, alcohol consumption and conduct problems were strongly associated with cannabis use. At age 21 years, compared with the non-user class, late-onset occasional, early-onset occasional and regular cannabis user classes had higher odds of nicotine dependence (OR=3.5, 95% CI 0.7 to 17.9; OR=12.1, 95% CI 1.0 to 150.3; and OR=37.2, 95% CI 9.5 to 144.8, respectively); harmful alcohol consumption (OR=2.6, 95% CI 1.5 to 4.3; OR=5.0, 95% CI 2.1 to 12.1; and OR=2.6, 95% CI 1.0 to 7.1, respectively); and other illicit drug use (OR=22.7, 95% CI 11.3 to 45.7; OR=15.9, 95% CI 3.9 to 64.4; and OR=47.9, 95% CI 47.9 to 337.0, respectively).

**Conclusions:**

One-fifth of the adolescents in our sample followed a pattern of occasional or regular cannabis use, and these young people were more likely to progress to harmful substance use behaviours in early adulthood.

## Introduction

Cannabis use is the commonest form of illicit substance use in the UK and many other countries^1^ ^2^ and is central to public and policy debate on risks and benefits of drug control, classification and harms.^3^ The potential harms of cannabis use during adolescence include altered brain development, cognitive impairment, chronic bronchitis and adverse mental health outcomes.^4^ Evidence on the role of adolescent cannabis use as a gateway to use of other illicit drugs, and its relationship with tobacco use and alcohol, is inconsistent.^5–12^

Longitudinal studies are required to tease out the relationship between cannabis use exposure and drug-related harm.^7^ ^13^ As young people do not initiate cannabis use at the same time, or develop similar patterns during adolescence, latent variable modelling is a useful tool to characterise different adolescent cannabis trajectories.^14^ ^15^ Further, to test the relationship between adolescent exposure and outcomes in adulthood, it is important that the trajectories are developed independently of the outcome.^16^ ^17^ Studies have developed joint trajectories of cannabis and other substances^18^ ^19^ but few have developed trajectories based on cannabis use alone. Those that have developed trajectories solely based on cannabis use tend to continue them well into adulthood and focus on a single outcome, rather than defining trajectories during adolescence in relation to multiple outcomes during early adulthood.^16^ ^17^ ^20^

In this paper, we aim to use data from the Avon Longitudinal Study of Parents and Children (ALSPAC) to (1) describe patterns of cannabis use during adolescence (rather than patterns of multiple substances) using longitudinal latent class analysis; (2) to determine factors associated with these patterns (including indicators of socioeconomic status); and (3) to investigate whether cannabis use predicts the use of tobacco, alcohol and other illicit drug use in early adulthood (age 21 years).

## Methods

### Study population

ALSPAC is a UK population-based birth cohort.^21^ Pregnant women residing in the former Avon Health Authority in south-west England who had an estimated date of delivery between 1 April 1991 and 31 December 1992 were invited to take part, resulting in a cohort of 14 541 pregnancies and 13 978 children alive at 12 months of age. Ethical approval for this study was obtained from the ALSPAC Law and Ethics Committee and the Local Research Ethics Committees. The ALSPAC study website contains details of all the data that are available through a fully searchable data dictionary (http://www.bris.ac.uk/alspac/researchers/data-access/data-dictionary/).

### Measures

#### Cannabis use

Information on cannabis use was collected on six occasions throughout adolescence via questionnaire (Q) or during a clinic (C). Median ages at response were: 13 years 10 months (C), 14 years 2 months (Q), 15 years 5 months (C), 16 years 7 months (Q), 17 years 9 months (C) and 18 years 8 months (Q). For simplicity, we will refer to these ages as 13, 14, 15, 16, 17 and 18 years. Responses to one or more questions at each time point were used to derive a repeated three-level ordinal variable with categories ‘Do not use’, ‘Occasional’ (typically less than once per week) and ‘Frequent’ (typically once a week or more) (full details in online supplementary table S1).

10.1136/jech-2016-208503.supp1 supplementary tables

#### Predictors

A range of predictors were considered as potential risk factors for trajectories of cannabis use. These measures have previously been shown to be associated with profiles of tobacco use^22^ and more modestly associated with profiles of alcohol use.^23^ Measures included (1) demographic variables collected prebirth around the time of enrolment, comprising housing tenure, crowding status, maternal education and parity; (2) maternal substance use in the offspring's later childhood collected via questionnaire, which comprised maternal smoking and alcohol consumption when the offspring were 12 years old, and maternal cannabis use when the offspring were aged 9 years, and (3) young person's factors collected through focus clinic at age 12 years 10 months and postal questionnaire at 11 years, which comprised tobacco/alcohol use at 12 years 10 months, and conduct problems at 11 years using the mother-reported Strengths and Difficulties Questionnaire.^24^

#### Problematic substance use behaviours at age 21 years

Data for outcome measures at age 21 (median 20 years 11 months) years were obtained via postal questionnaire. We used a cut-off of 16 points and above on the alcohol use disorders identification test^13^ ^25^ to indicate ‘harmful’ alcohol use, and compared this with ‘low-risk’ or ‘hazardous’ as a combined reference group. We used the Fagerström test for nicotine dependence,^26^ comparing medium/high/very high nicotine dependence with non-smoker/very low/low dependence.

We derived an indicator for those reporting the use of any of the following other illicit drugs (except cannabis) in the previous 3 months. Of those reporting recent use (N=462), 176 (38%) had used cocaine, 278 (60%) had used amphetamine, 136 (30%) had used inhalants, 72 (16%) had used sedatives, 105 (23%) had used hallucinogens and 25 (6%) had used opioids. We did not use a measure of other illicit drug abuse or dependence due to the low prevalence in our sample. As initiation of substance use is implicated as triggering the cascade in the gateway hypothesis,^5^ any use of other illicit drugs could be regarded as problematic or worthy of concern.

### Statistical methods

Longitudinal latent class analysis was used to derive trajectories of cannabis use. To establish the optimal number of latent classes, we used: (1) the sample size-adjusted Bayesian information criterion; (2) the bootstrap likelihood ratio test;^27^ (3) entropy and (d) bivariate model fit information. We repeated the estimation procedure while varying the amount of missing data. Analyses were carried out using Mplus V.7.11.^28^ More information is provided in online supplementary table S2.

Risk factors for class membership were estimated using multinomial logistic regression models using the normative latent class as the baseline category for the outcome before reparameterising to derive comparisons across the other outcome classes. Parameter estimates were obtained using the ‘Modal ML’ three-step method.^29^ ^30^ This has been shown to produce less biased estimates than traditional three-step ‘classify–analyse’ methods while avoiding the problem of covariates impacting on the measurement model itself.^29^

The association between latent class membership and subsequent harmful behaviours at age 21 years was estimated using logistic regression models with class membership as a nominal predictor (also employing the modal ML technique). Estimates were adjusted for the potential confounding effects of: sex; sociodemographic indicators, maternal substance use; child's conduct problems in late childhood; and finally alcohol and tobacco use at age 12 years, 10 months. There were insufficient cases of other illicit drug use at this earlier age, so these data were not used.

## Results

Of 13 978 children in ALSPAC, the sample with available data ranged from 4664 at age 13 to 2939 at age 18 years. A total of 5315 (38.0%) adolescents had cannabis use assessed at three or more time points, of whom 2921 (55.0%) had complete data for all covariates and 1571 (29.6%) had data for all covariates and substance use outcomes at age 21 years. Differences between participants with and without complete data were consistent with those shown previously,^31^ with higher losses to follow-up among families from lower social classes (data not shown).

### Patterns of cannabis use

**Figure 1:**
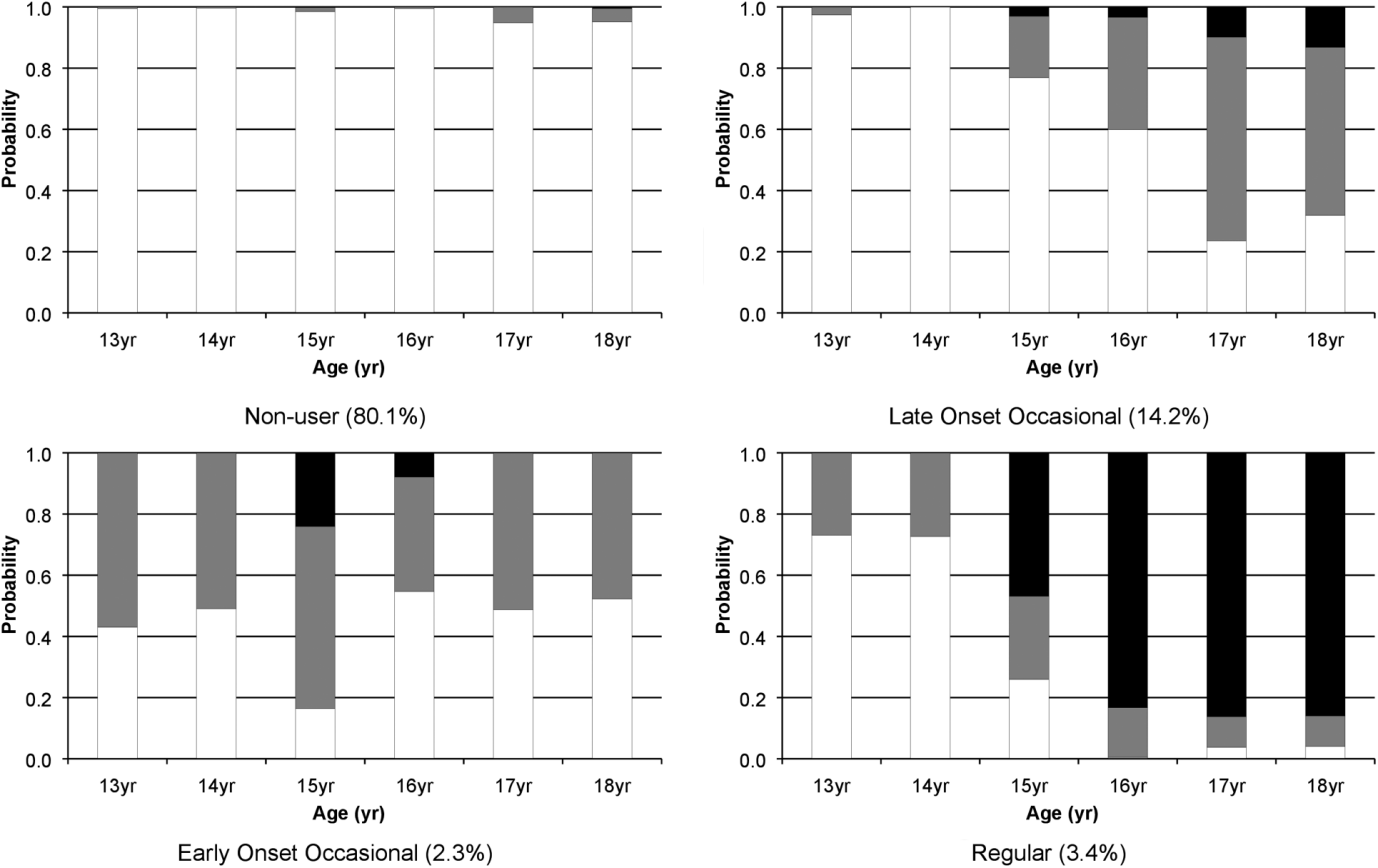
Profiles of cannabis use from latent class analysis (n=5315). Key: black shading = regular cannabis use; grey = occasional cannabis use; white = no current cannabis use.

The prevalence of both occasional and regular cannabis use increased between the ages of 13 and 18 years (table 1). There was good agreement that a four-class solution was adequate in explaining the heterogeneity in the cannabis use data. Much of the gains made by using the sample with one or more measures were lost following the inclusion of the early risk factors. As such, we focus on the sample for which 3+ cannabis use measurements were available (model fit statistics and a discussion of our decision process can be found in online supplementary table S2). This four-class solution comprised patterns of cannabis use that we labelled as ‘non-user’ (80.1%), ‘late-onset occasional’ (14.2%), ‘early-onset occasional’ (2.3%) and ‘regular user’ (3.4%) (figure 1).

**Table 1:** Prevalence of cannabis use at each time point estimated using all available data

	13 years	14 years	15 years	16 years	17 years	18 years
	n=4664	n=4571	n=4427	n=4196	n=3550	n=2939
Do not use	4525 (97.0%)	4470 (97.8%)	4032 (91.1%)	3782 (90.1%)	2868 (80.8%)	2457 (83.6%)
Occasional	139 (3.0%)	101 (2.2%)	275 (6.2%)	284 (6.8%)	533 (15.0%)	353 (12.0%)
Frequent	0 (0.0%)	0 (0.0%)	120 (2.7%)	130 (3.1%)	149 (4.2%)	129 (4.4%)

Derivation of the three categories used here is provided in online supplementary table S1.

### Risk factors associated with patterns of cannabis use

Household income and factors indicative of lower socioeconomic status were neither strong nor consistently associated with cannabis use. Living in rented or subsidised housing, maternal tobacco smoking when the child was 12 years old and childhood (12 years and 10 months) substance use and conduct problems were associated with regular cannabis use. Childhood tobacco and alcohol use prior to cannabis trajectories showed the greatest association with early-onset occasional cannabis use. Being female was inversely associated with regular cannabis use. Having an older sibling was associated with higher odds of early-onset occasional cannabis use. Lower levels of maternal education were associated with lower odds of late-onset and-early onset occasional cannabis use. Mother’s use of cannabis when the child was 9 years old was associated with late-onset occasional, early-onset occasional and regular cannabis use (table 2).

**Table 2:** Factors associated with cannabis use latent class membership

Covariate	Late-onset occasional OR (95% CI)	Early-onset occasional OR (95% CI)	Regular OR (95% CI)	p Value
Sex				
Male	1.00 ref			<0.001
Female	0.86 (0.69 to 1.08)	0.94 (0.56 to 1.58)	0.32 (0.22 to 0.48)	
Housing tenure				
Mortgaged/owned home	1.00 ref			<0.001
Rented	1.52 (1.04 to 2.23)	0.87 (0.27 to 2.84)	1.76 (0.98 to 3.16)	
Subsidised rented	0.89 (0.54 to 1.46)	1.10 (0.39 to 3.10)	2.32 (1.39 to 3.87)	
Sibling order				
First child	1.00 ref			<0.001
Second child	1.11 (0.85 to 1.43)	2.00 (1.03 to 3.86)	1.27 (0.85 to 1.88)	
Third child or higher	1.35 (0.98 to 1.85)	3.53 (1.77 to 7.01)	1.24 (0.73 to 2.10)	
Home overcrowding				
Up to one person per room	1.00 ref			0.21
>1 person per room	2.16 (1.29 to 3.63)	0.70 (0.08 to 6.19)	1.32 (0.47 to 3.71)	
Maternal education				
Qualifications beyond high school	1.00 ref			<0.001
High school qualifications	0.54 (0.41 to 0.71)	0.53 (0.29 to 0.96)	0.94 (0.63 to 1.41)	
No high school qualifications	0.53 (0.37 to 0.75)	0.28 (0.10 to 0.81)	1.02 (0.63 to 1.63)	
Household income				
High (top quintile)	1.00 ref			<0.001
Middle high	0.64 (0.45 to 0.92)	1.28 (0.65 to 2.52)	0.36 (0.17 to 0.76)	
Middle	0.79 (0.56 to 1.11)	0.67 (0.27 to 1.62)	1.14 (0.68 to 1.90)	
Middle low	0.75 (0.52 to 1.08)	1.02 (0.46 to 2.23)	1.08 (0.63 to 1.86)	
Low (bottom quintile)	0.77 (0.52 to 1.15)	0.44 (0.13 to 1.54)	1.08 (0.61 to 1.94)	
Maternal alcohol use when child aged 12				
<14 units per week	1.00 ref			0.003
14+ units per week	1.93 (1.49 to 2.49)	2.57 (1.47 to 4.52)	1.59 (1.05 to 2.40)	
Maternal alcohol binge when child aged 12				
No	1.00 ref			<0.001
Yes	1.58 (1.22 to 2.04)	2.08 (1.18 to 3.64)	1.51 (1.01 to 2.27)	
Maternal smoking when child aged 12				
No	1.00 ref			0.001
Yes	1.30 (0.91 to 1.86)	2.60 (1.42 to 4.78)	2.92 (1.89 to 4.51)	
Maternal cannabis use when child aged 9				
No	1.00 ref			<0.001
Yes	3.23 (1.87 to 5.56)	9.38 (4.37 to 20.2)	11.3 (6.88 to 18.7)	
Child smoking at 13 years				
No	1.00 ref			<0.001
Yes	2.15 (1.39 to 3.33)	15.2 (8.69 to 26.5)	10.5 (6.96 to 16.0)	
Child drinking alcohol at 13 years				
No	1.00 ref			<0.001
Less than weekly	2.43 (1.75 to 3.37)	7.89 (3.44 to 18.1)	1.77 (0.90 to 3.46)	
Weekly	2.30 (1.47 to 3.60)	25.0 (11.9 to 52.4)	7.49 (4.72 to 11.9)	
Child conduct problems at 11 years				
Low	1.00 ref			<0.001
Medium	1.40 (1.06 to 1.84)	1.43 (0.75 to 2.73)	2.12 (1.37 to 3.28)	
High	1.39 (0.80 to 2.41)	3.61 (1.61 to 8.12)	5.60 (3.30 to 9.50)	

Estimates shown are multinomial ORs estimated using all available data for each covariate and ‘Non-user’ as the reference category for the cannabis use outcome. Omnibus p values are from Wald tests examining the overall association between each risk factor and cannabis use latent class membership.

Alcohol binge defined as 4+ units of alcohol on one occasion. One unit of alcohol defined as 0.8 g ethanol.

### Associations of cannabis use with harmful behaviours at age 21 years

Cannabis use during adolescence was associated with increased odds of harmful substance use behaviours at age 21 years, compared with non-users (table 3). There was evidence for an association between cannabis use and nicotine dependence, with stronger associations being observed with increasing cannabis use latent classes (ie, consistent with a dose–response relationship). Regular cannabis use showed the strongest association with other illicit drug use. Early-onset cannabis use (in comparison to no cannabis use) showed the strongest association with harmful alcohol consumption, with late-onset cannabis use and regular cannabis use also being associated. When assessing cannabis as a risk factor for nicotine dependence and illicit drug use, an increase in risk between cannabis classes was also observed when comparing regular use to early-onset and late-onset cannabis use, respectively.

**Table 3:** Associations of cannabis use latent class membership with harmful behaviours at 21 years

		Unadjusted models	Adjusted models 1	Adjusted models 2	Adjusted models 3	Adjusted models 4
	Reference class	OR (95% CI)	OR (95% CI)	OR (95% CI)	OR (95% CI)	OR (95% CI)
*Nicotine dependence*		n=3215	n=2772	n=2197	n=2134	n=1863
Late-onset occasional	Non-user	4.43 (1.82 to 10.8)	3.68 (0.98 to 13.9)	2.52 (0.38 to 16.9)	2.58 (0.44 to 15.1)	3.54 (0.70 to 17.9)
Early-onset occasional	Non-user	5.78 (1.02 to 32.8)	7.66 (0.78 to 74.9)	13.5 (2.02 to 90.0)	9.81 (1.25 to 76.6)	12.1 (0.97 to 150.3)
Regular	Non-user	26.7 (12.3 to 57.9)	60.6 (22.8 to 161.3)	51.1 (14.2 to 183.3)	37.1 (11.0 to 125.2)	37.2 (9.53 to 144.8)
Early-onset occasional	Late-onset occasional	1.30 (0.20 to 8.48)	2.08 (0.15 to 28.4)	5.33 (0.38 to 74.6)	3.80 (0.28 to 52.2)	3.41 (0.18 to 66.2)
Regular	Late-onset occasional	6.03 (2.30 to 15.8)	16.5 (3.89 to 69.9)	20.2 (2.19 to 186.7)	14.4 (1.94 to 106.3)	10.5 (1.42 to 77.7)
Regular	Early-onset occasional	4.62 (0.72 to 29.7)	7.92 (0.76 to 82.4)	3.79 (0.54 to 26.71)	3.78 (0.42 to 34.1)	3.08 (0.24 to 40.1)
*Harmful alcohol consumption*		n=3046	n=2631	n=2093	n=2034	n=1772
Late-onset occasional	Non-user	4.26 (2.96 to 6.13)	3.93 (2.65 to 5.83)	3.02 (1.90 to 4.82)	3.09 (1.92 to 4.97)	2.59 (1.54 to 4.34)
Early-onset occasional	Non-user	8.18 (3.99 to 16.8)	8.53 (4.09 to 17.8)	7.34 (3.33 to 16.2)	7.68 (3.47 to 17.0)	5.03 (2.09 to 12.1)
Regular	Non-user	3.45 (1.78 to 6.69)	2.63 (1.23 to 5.64)	2.67 (1.13 to 6.31)	2.95 (1.22 to 7.11)	2.61 (0.96 to 7.08)
Early-onset occasional	Late-onset occasional	1.92 (0.87 to 4.24)	2.17 (0.96 to 4.89)	2.43 (1.00 to 5.91)	2.48 (1.02 to 6.04)	1.95 (0.73 to 5.19)
Regular	Late-onset occasional	0.81 (0.39 to 1.70)	0.67 (0.29 to 1.55)	0.88 (0.34 to 2.27)	0.95 (0.36 to 2.49)	1.01 (0.34 to 2.98)
Regular	Early-onset occasional	0.42 (0.16 to 1.15)	0.31 (0.11 to 0.90)	0.36 (0.11 to 1.16)	0.38 (0.12 to 1.24)	0.52 (0.14 to 1.86)
*Other Illicit drug use*		n=3048	n=2631	n=2094	n=2033	n=1772
Late-onset occasional	Non-user	13.1 (8.79 to 19.4)	13.8 (8.93 to 21.3)	10.8 (6.59 to 17.7)	11.3 (6.85 to 18.8)	8.47 (4.99 to 14.4)
Early-onset occasional	Non-user	8.26 (3.75 to 18.2)	6.64 (2.59 to 17.0)	3.34 (1.03 to 10.8)	3.41 (1.06 to 10.92)	3.21 (0.85 to 12.1)
Regular	Non-user	27.0 (14.2 to 51.5)	28.7 (13.55 to 60.6)	25.6 (8.97 to 73.1)	29.1 (8.49 to 100.0)	25.9 (7.13 to 94.0)
Early-onset occasional	Late-onset occasional	0.63 (0.27 to 1.48)	0.48 (0.18 to 1.32)	0.31 (0.09 to 1.07)	0.30 (0.09 to 1.03)	0.38 (0.09 to 1.51)
Regular	Late-onset occasional	2.07 (1.02 to 4.21)	2.08 (0.93 to 4.67)	2.37 (0.74 to 7.53)	2.57 (0.67 to 9.86)	3.06 (0.77 to 12.1)
Regular	Early-onset occasional	3.27 (1.17 to 9.14)	4.31 (1.30 to 14.4)	7.67 (1.55 to 37.9)	8.55 (1.51 to 48.4)	8.08 (1.26 to 51.6)

Rates of harmful behaviours across class were as follows: non-cannabis users (1% nicotine dependence, 8.4% harmful alcohol consumption, 13.6% other illicit drug use); late-onset occasional cannabis users (4.3% nicotine dependence, 28.0% harmful alcohol consumption, 83.1% other illicit drug use); early-onset occasional cannabis users (5.5% nicotine dependence, 42.7% harmful alcohol consumption, 83.0% other illicit drug use); regular cannabis users (21.2% nicotine dependence, 23.9% harmful alcohol consumption, 94.3% other illicit drug use).

Reference categories for outcomes are: nicotine dependence ‘non-smoker/low/very low dependence’; alcohol consumption ‘low risk/hazardous’; other illicit drug use ‘never used/not used in last 3 months’.

Confounders adjusted for: (model 1) sex; household income, housing tenure; crowding status; birth order; maternal educational attainment; (model 2) additionally adjusted for maternal substance use (smoking, alcohol consumption and cannabis use); (model 3) additionally adjusted for child conduct problems at age 11 years; (model 4) additionally adjusted for tobacco and alcohol use at age 13 years.

All p values <0.001.

Adjustment for potential confounders showed evidence of negative and positive confounding. Sociodemographic measures negatively confounded the crude associations of cannabis use with nicotine dependence, slightly positively confounded the crude associations of cannabis use with harmful alcohol consumption and had little or no confounding effect on crude associations of cannabis use with other illicit drug use. Unadjusted estimates from the complete case sample for each adjusted model showed that substantial changes to the adjusted estimates were the result of adjustment for confounders and not selection bias in the decreased sample (see online supplementary table S3). Further investigation showed inconsistent social patterning across the three outcomes considered (see online supplementary table S4), with low maternal education and low household income being positively associated with nicotine dependence yet inversely associated with alcohol and other illicit drug use.

## Discussion

We characterised four adolescent cannabis use trajectories—with ∼80% classified as non-users, 17% as infrequent users (late or early-onset) and over 3% as regular users. Males were more likely to belong to the regular user class. We found a strong positive association between the cannabis use trajectories and harmful substance use in adulthood. In adjusted models, regular adolescent cannabis users had 37-fold, 3-fold and 26-fold higher odds of tobacco dependence, harmful alcohol use and other illicit drug use, respectively, in adulthood compared with non-users. Early-onset and late-onset occasional adolescent cannabis classes also were associated with these outcomes, with evidence for a dose–response effect observed between cannabis use and tobacco dependence. Cannabis use trajectories were associated with maternal substance use, child conduct problems and early tobacco or alcohol use. We observed a moderate to strong effect of social patterning on the cannabis use trajectories.

### Strengths and limitations

ALSPAC is a well-characterised birth cohort with repeated measures of cannabis use—which has been used in several other studies of adolescent substance use.^22^ ^23^ The number of people analysed in our study is larger than many other studies including a recent synthesis of three cohorts,^32^ and our estimates are consistent with household and school surveys.^33^ However, there are limitations to our study.

First, there are considerable losses to follow-up—with high rates of attrition among less affluent families and participants who may be more likely to use cannabis and adopt harmful behaviours in early adulthood. We have previously observed differences in the prevalence of atypical trajectories of smoking behaviour, with twice the rate of persistent daily users derived following the inclusion of partial responders,^22^ but not in alcohol trajectories.^23^ On this occasion, we observe more moderate changes in the class distribution when altering the analytical sample, which is likely to be a reflection of the fact that in our UK birth cohort, cannabis use is less socially patterned than tobacco use. Although the proportions of regular and early-onset users decreased slightly as the number of time points increased (regular users from 3% with 3+ time points to 2% with all 6 time points, early-onset occasional users from 2% to 1%), the overall distribution (∼80% non-users, ∼15% late-onset occasional users, ∼5% regular or early-onset users) remained stable (see online supplementary table S2). With regard to missing covariate information, listwise deletion remains the status quo^34^ with methodological obstacles to the application of alternatives. There is currently a lack of research on the use of other methods, such as multiple imputation, for latent class analysis.^35^ ^36^ Standard imputation methods are ineffective when group membership is inferred from the data (ie, is latent) as group differences in the mean and covariance structure across the hidden latent classes will not be preserved. This has previously been demonstrated and discussed in detail.^36^ In brief, when using multiple imputation in this framework, we would effectively be conducting an imputation under a multigroups SEM model, where the grouping is unknown. In addition, the effect sizes for tobacco dependence and other illicit drug use are unlikely to be reversed or negated by any analysis on imputed data.

Second, the potential contribution made by concurrent use of other substances and other conditions that often co-occur with cannabis warrants careful consideration. We have shown strong associations between patterns of adolescent cannabis use and problematic use of other substances in early adulthood. When studying cannabis and risk of, for example, harmful alcohol use, it is possible that some of the observed association between cannabis use and alcohol use is due to the emergence of alcohol use concurrent to the use of cannabis. Previous literature has reported that studies of longitudinal psychosocial outcomes^37^ should strive to better address the potential problem of confounding. While we are not addressing psychosocial outcomes here, we acknowledge that this is a transferable issue. Owing to our chosen longitudinal mixture model, we have opted to adjust for early use of alcohol and tobacco, that is, measured before cannabis use was first considered. We have previously shown these measures to be strongly predictive of subsequent use,^22^ ^23^ and we also observe early alcohol/tobacco use to confer a considerable risk of our 21-year problem outcomes (see online supplementary table S5).

Finally, our data on exposure and outcome are based on self-report and so maybe subject to misclassification. However, for population surveys there are no reliable biological alternatives for cannabis or alcohol.^38^ ^39^

### Findings in context with other studies and implications

We find different effects of social patterning on substance use trajectories. Maternal education was observed to have a negative impact on cannabis in contrast to a moderate effect of social patterning on alcohol trajectories and strong effect on tobacco trajectories.^22^ ^23^ This provides evidence that our findings cannot be solely due to residual confounding due to poorly measured social factors, since these social factors are differently associated with each 21-year outcome.

Other studies of latent class trajectories of cannabis use in adolescence have reported similar numbers of group trajectories with similar definitions and sizes.^14^ ^17^ ^20^ As with other studies, our trajectories did discriminate clearly between participants, and could be used as ‘categorical’ variables rather than distributions.^16–18^ Few longitudinal studies have solely examined cannabis use across adolescence and its relationship with other drug behaviours in early adulthood. Other studies have sought to describe trajectories using a composite measure of two or more drugs—or extended the trajectories into later ages when the outcome occurs and so are not directly comparable with our study. As we have shown differences in social patterning for different licit and illicit substances, a single composite measure of drug use does not seem appropriate for this population.

Previous studies have used a wide range of techniques to provide evidence for or against the gateway hypothesis including standard epidemiological techniques,^11^ ^12^ co-twin analysis,^8^ longitudinal modelling,^7^ genetic studies^9^ and mixed models.^10^ Our finding that regular cannabis users have greater odds of later nicotine dependence, harmful alcohol use and other illicit drug use is consistent with many other studies that have sought to examine the gateway hypothesis.^6^ ^8–10^ Here, we have observed stronger associations than those generally reported, which may be in part because our outcomes are more refined (at age 21) than other studies that have examined cannabis use at a single time point or measure the outcome many years after the trajectories.^20^ Several other studies report either associations between cannabis exposure or dependence and other illicit drug use,^17^ ^18^ or alcohol consumption^20^ whereas we assess associations between licit and illicit behaviours, including nicotine dependence which, to the best of our knowledge, has not previously been assessed. However, our results are inconsistent with some studies that have reported no association between cannabis use and alcohol use^7^ or between cannabis use and tobacco use.^11^

Interpretation of the underlying mechanism of the association, however, cannot be tested by our analyses. Our data are consistent with adolescent cannabis use as a ‘gateway’ to adult substance dependence and illicit drug use which could relate either to a biological, behavioural or environmental mechanism; and our data are consistent with theories of shared genetic vulnerabilities to substance use.^40^ Equally, we cannot entirely rule out confounding as an alternative explanation. Cannabis and tobacco are highly correlated and it is difficult to distinguish their separate effects. Furthermore, the gateway hypothesis suggests a sequence to the use of various drugs, as such there is no single ‘gateway’ drug and one might expect tobacco and alcohol to predict later cannabis use and other illicit drug use.^22^ ^23^

### Conclusion

Adolescent substance use also clusters with other risk behaviours which are strongly associated with outcomes in adulthood. Our study does not support or refute arguments for altering the legal status of cannabis use—especially since two of the outcomes are legal in the UK. This study and others do, however, lend support to public health strategies and interventions that aim to reduce cannabis exposure in young people.

**What is already known on this subject?**

- Different forms of substance use in adolescents and young people cluster.
- However, support for the ‘gateway hypothesis’ suggesting that cannabis leads to the use of other substances is inconsistent.
- In part, this is because of a lack of longitudinal studies and refined measures of cannabis exposure during adolescence.

**What this study adds?**

- Robust cannabis use trajectories were defined and categorised as ‘non-users’ (80.1%), ‘late-onset occasional’ (14.2%), ‘early-onset occasional’ (2.3%) and ‘regular’ users (3.4%).
- There was a dose–response relationship between cannabis use trajectories in adolescence and nicotine dependence, harmful alcohol consumption and other illicit drug use by age 21.
