# Supplementary material for: Patterns of cannabis use during adolescence and their association with harmful substance use behaviour: findings from a UK birth cohort
Source: J Epidemiol Community Health. 2017 Jun 7;71(8):764–70. doi: 10.1136/jech-2016-208503 (PMC5537531; doi:10.1136/jech-2016-208503)
Supplement: supplementary tables [file jech-2016-208503supp001.pdf]

Table S1: Recoding of cannabis-use questions at each time-point into variable on which latent class analysis was based

Age<br>Source	13 years<br>Computer in clinic	14 years<br>Postal questionnaire	15 years<br>Computer in clinic	16 years<br>Postal questionnaire	17 years<br>Computer in clinic	18 years<br>Postal questionnaire
Do not use	“Have you ever tried cannabis?” = “No”	“Have you ever tried cannabis?” = “No”<br>Or, if “yes” to stem, “I have only ever tried cannabis once or twice” or “I used to sometimes use or take cannabis but I never do now”	“Have you ever tried cannabis?” = “No”<br>Or, if “yes” to stem, “I have only ever tried cannabis once or twice” or “I used to sometimes use or take cannabis but I never do now”	“Have you ever tried cannabis?” = “No”<br>Or, if “yes” to stem, “I have only ever tried cannabis once or twice” or “I used to sometimes use or take cannabis but I never do now”	Ever tried cannabis? = “No”	“Have you ever tried cannabis?” = “No”
Occasional	If “yes” to stem, “How many times in the last 6 months?” = “1-3 times” or “>4 times”	If “yes” to stem, “I sometimes use or take cannabis but less often than once a week”	If “yes” to stem, “I sometimes take cannabis but less often than once a week”	If “yes” to stem, “I sometimes use or take cannabis but less often than once a week”	If “yes” to stem, cannabis use in past 12 months = “Monthly or less” or “2-4 times per month”	If “yes” to stem, “In the last 12 months, how often have you used cannabis?” = “Once or twice” or “Less than monthly” or “Monthly (but less than weekly)”
Frequent	If “yes” to stem, “How many times in the last 6 months?” = “1 x per week”	If “yes” to stem, “I usually use or take cannabis between one and six times a week” or “I usually use or take cannabis more than six times a week, but I don't use it every day” or “I usually use or take cannabis every day”	If “yes” to stem, “I usually use or take cannabis between one and six times a week” or “I usually use or take cannabis more than six times a week, but I don't use it every day” or “I usually use or take cannabis every day”	If “yes” to stem, “I usually use or take cannabis between one and six times a week” or “I usually use or take cannabis more than six times a week, but I don't use it every day” or “I usually use or take cannabis every day”	If “yes” to stem, cannabis use in past 12 months = “2-3 times per week” or “4+ times per week”	If “yes” to stem, “In the last 12 months, how often have you used cannabis?” = “Weekly” or “Daily or almost daily”

Table S2: Model fit statistics for cannabis-use latent class models

	Number of classes (c)					
	2	3	4	5	6	7
			1+ measures (n=8,103)			
SSA-BIC	16445.9	16230.2	16178.2	16230.2	16283.7	16353.8
Conditional independence	1896.3	567.0	158.9	116.0	83.3	87.0
Entropy	0.766	0.734	0.765	0.780	0.743	0.755
LMR p-value	< 0.001	< 0.001	< 0.001	< 0.001	1.000	0.563
BLRT p-value	< 0.001	< 0.001	< 0.001	0.072	0.006	0.608
Smallest class size	1168	324	235	98	115	65
			3+ measures (n=5,315)			
SSA-BIC	13748.5	13492.1	13402.7	13411.8	13425.1	13456.3
Conditional independence	1207.4	331.1	92.0	65.5	34.2	33.6
Entropy	0.848	0.809	0.833	0.843	0.806	0.815
LMR p-value	< 0.001	< 0.001	< 0.001	0.051	0.283	0.718
BLRT p-value	< 0.001	< 0.001	< 0.001	0.208	0.766	0.660
Smallest class size	733	184	121	51	60	36
			All 6 measures (n=1,481)			
SSA-BIC	4535.8	4449.9	4451.3	4493.5	4543.2	4600.3
Conditional independence	439.2	139.3	32.3	22.2	18.2	11.4
Entropy	0.890	0.887	0.905	0.904	0.906	0.928
LMR p-value	< 0.001	0.222	< 0.001	0.002	0.145	0.446
BLRT p-value	< 0.001	< 0.001	< 0.001	< 0.001	0.006	0.032
Smallest class size	201	31	17	5	6	7

SSA-BIC - sample-size adjusted Bayesian Information Criterion. The Bayesian Information Criterion (BIC) is the traditional fit statistic for comparing mixture models. BIC will typically decrease and then increase following the incremental additional of classes. Using this statistic the model with the lowest BIC (or other models with BIC values in that vicinity) would be deemed optimal. There is good support for 4-classes using the BIC. The 3-class model is deemed optimal for the complete case sample (n = 1,481) but this is to be expected given the smaller sample size.

Conditional independence. This is an assessment of the remaining association between each pair of measurements once heterogeneity accounted for by latent C has been removed. There is currently no accepted threshold for this measure however we tend to observe marked improvements (reductions) followed by smaller changes. Here these data suggests improvements up until 6 classes.

Entropy. This is a measure of classification accuracy, and whilst it is generally of little use in determining the optimal model, it indicates the level of bias which one would expect were a standard three-step estimation to be performed. Here entropy is fairly high and above the commonly-used threshold of 0.8 for all models. We have recently demonstrated (paper in press) that bias can result even for values reaching 0.9 and hence we have opted for the bias-adjusted three-step method as described in the methods.

The Bootstrap Likelihood Ratio Test (BLRT) and the Lo-Mendell-Rubin (LMR) test statistics both assess change in model fit when adding an additional class. Here a high p-value for a k-class model indicates no substantial improvement in fit compared to the k-1 class solution. Whilst the BLRT is usually more conservative, both are in support of a 4-class solution for the 3+ measurement sample.

Other criteria. The five-class solution was differentiated from the four-class solution by separation of regular users into early and late-onset and a substantial reduction in the size of the smallest class.

Change in class distribution: The class distribution across the latent classes using varying amounts of missing data was as followings for 1+, 3+ and 6 measures, respectively: Regular users: 4%, 3%, 2%; late onset occasion users: 14%, 14%, 15%; early-onset occasional users: 3%, 2%, 1%; non-users: 80%, 80%, 82%.

Table S3. Unadjusted associations across increasingly restricted samples

	Reference class	Sample for unadjusted models OR (95% CI)	Sample for adjusted models 1 OR (95% CI)	Sample for adjusted models 2 OR (95% CI)	Sample for adjusted models 3 OR (95% CI)	Sample for adjusted models 4 OR (95% CI)
Nicotine dependence		n=3,215	n=2,772	n=2,197	n=2,134	n=1,863
Late onset occasional	Non-user	4.43 [1.82, 10.8]	3.29 [1.02, 10.6]	2.44 [0.52, 11.4]	2.51 [0.56, 11.3]	2.87 [0.67, 12.3]
Early onset occasional	Non-user	5.78 [1.02, 32.8]	4.97 [0.50, 49.1]	8.31 [1.21, 57.3]	8.30 [1.27, 54.4]	10.1 [1.65, 61.6]
Regular	Non-user	26.7 [12.3, 57.9]	35.1 [14.9, 82.7]	28.8 [10.0, 82.6]	24.4 [8.06, 74.1]	17.9 [4.82, 66.4]
Early onset occasional	Late onset occasional	1.30 [0.20, 8.48]	1.51 [0.12, 18.7]	3.41 [0.31, 37.6]	3.31 [0.32, 34.0]	3.51 [0.39, 31.8]
Regular	Late onset occasional	6.03 [2.30, 15.8]	10.7 [3.18, 35.8]	11.8 [2.30, 60.6]	9.75 [1.88, 50.5]	6.24 [1.11, 35.2]
Regular	Early onset occasional	4.62 [0.72, 29.7]	7.07 [0.64, 78.6]	3.46 [0.42, 28.7]	2.94 [0.36, 24.0]	1.78 [0.21, 15.2]
Harmful alcohol consumption		n=3,046	n=2,631	n=2,093	n=2,034	n=1,772
Late onset occasional	Non-user	4.26 [2.96, 6.13]	4.10 [2.79, 6.03]	3.49 [2.24, 5.42]	3.56 [2.27, 5.58]	3.29 [2.03, 5.32]
Early onset occasional	Non-user	8.18 [3.99, 16.8]	8.46 [3.91, 18.4]	8.35 [3.57, 19.5]	8.53 [3.67, 19.9]	7.16 [2.86, 17.9]
Regular	Non-user	3.45 [1.78, 6.69]	3.07 [1.45, 6.52]	3.51 [1.52, 8.12]	3.90 [1.68, 9.08]	4.27 [1.69, 10.8]
Early onset occasional	Late onset occasional	1.92 [0.87, 4.24]	2.06 [0.88, 4.83]	2.40 [0.93, 6.16]	2.40 [0.93, 6.15]	2.18 [0.78, 6.07]
Regular	Late onset occasional	0.81 [0.39, 1.70]	0.75 [0.33, 1.72]	1.01 [0.40, 2.57]	1.10 [0.43, 2.81]	1.30 [0.46, 3.66]
Regular	Early onset occasional	0.42 [0.16, 1.15]	0.36 [0.12, 1.10]	0.42 [0.12, 1.43]	0.46 [0.13, 1.56]	0.60 [0.16, 2.29]
Other illicit drug use		n=3,048	n=2,631	n=2,094	n=2,033	n=1,772
Late onset occasional	Non-user	13.1 [8.79, 19.4]	14.3 [9.35, 21.9]	12.7 [7.95, 20.2]	13.4 [8.30, 21.5]	10.8 [6.50, 17.8]
Early onset occasional	Non-user	8.26 [3.75, 18.2]	7.49 [3.11, 18.0]	5.25 [1.86, 14.9]	5.38 [1.92, 15.1]	6.27 [2.30, 17.1]
Regular	Non-user	27.0 [14.2, 51.5]	31.2 [14.8, 65.6]	36.4 [14.2, 93.2]	38.7 [14.4, 104]	40.4 [12.4, 131]
Early onset occasional	Late onset occasional	0.63 [0.27, 1.48]	0.52 [0.21, 1.33]	0.41 [0.14, 1.24]	0.40 [0.14, 1.20]	0.58 [0.20, 1.71]
Regular	Late onset occasional	2.07 [1.02, 4.21]	2.18 [0.97, 4.90]	2.87 [1.04, 7.88]	2.90 [1.01, 8.33]	3.75 [1.07, 13.2]
Regular	Early onset occasional	3.27 [1.17, 9.14]	4.17 [1.31, 13.3]	6.92 [1.67, 28.7]	7.20 [1.69, 30.6]	6.43 [1.34, 31.0]

Reference categories for outcomes are: nicotine dependence ‘non-smoker/low/very low dependence’; alcohol consumption ‘low risk/hazardous’; other illicit drug use ‘never used/not used in last 3mn’

model 1: sample restricted to those with sex; household income, housing tenure; crowding status; birth order; maternal educational attainment,

model 2: sample additionally restricted to those with maternal substance use information (smoking, alcohol consumption and cannabis-use);

model 3: sample additionally restricted to those with data on child conduct problems at age 11 years

model 4: sample additionally restricted to those with data on tobacco and alcohol use at age 13 years.

All p-values < 0.001

Table S4. Social patterning of the three 21-year outcomes

	Nicotine dependence		Harmful alcohol consumption		Other illicit drug use	
	% with outcome	OR [95% CI]	% with outcome	OR [95% CI]	% with outcome	OR [95% CI]
Sex						
Male	1.9%	1.00 ref.	15.8%	1.00 ref.	30.9%	1.00 ref.
Female	2.1%	1.13 [0.68, 1.89]	10.2 %	0.60 [0.49, 0.75]	22.5%	0.65 [0.55, 0.77]
		p=0.64		p<0.001		p<0.001
Housing tenure						
Mortgaged/owned home	1.8%	1.00 ref.	12.7%	1.00 ref.	25.7%	1.00 ref.
Rented	3.4%	1.95 [0.91, 4.16]	12.6%	0.98 [0.65, 1.49]	27.6%	1.10 [0.81, 1.50]
Subsidized rented	2.4%	1.35 [0.48, 3.78]	9.7%	0.73 [0.43, 1.27]	25.3%	0.98 [0.67, 1.42]
		p=0.20		p=0.54		p=0.82
Sibling order						
First child	1.9%	1.00 ref.	12.8%	1.00 ref.	24.1%	1.00 ref.
Second child	1.6%	0.81 [0.45, 1.48]	12.9%	1.01 [0.80, 1.28]	27.0%	1.16 [0.97, 1.40]
Third child or higher	2.8%	1.49 [0.78, 2.83]	10.6%	0.81 [0.58, 1.13]	28.2%	1.24 [0.98, 1.57]
		p=0.24		p=0.42		p=0.11
Home over-crowding						
≤ 1 person / room	1.9%	1.00 ref.	12.7%	1.00 ref.	25.8%	1.00 ref.
> 1 person / room	3.6%	1.92 [0.59, 6.26]	7.9%	0.59 [0.25, 1.37]	22.4%	0.83 [0.48, 1.43]
		p=0.27		p=0.21		p=0.50
Maternal education						
Qualifications beyond high school	1.6%	1.00 ref.	14.4%	1.00 ref.	30.1%	1.00 ref.
High school qualifications	1.8%	1.12 [0.62, 2.03]	11.4%	0.77 [0.60, 0.98]	21.9%	0.65 [0.54, 0.79]
No high school qualifications	3.5%	2.16 [1.17, 3.97]	8.6%	0.56 [0.40, 0.80]	20.3%	0.59 [0.46, 0.76]
		p=0.03		p=0.002		p<0.001
Household income						
High (top quintile)	1.4%	1.00 ref.	15.3%	1.00 ref.	29.8%	1.00 ref.
Middle high	1.3%	0.90 [0.37, 2.20]	12.7%	0.80 [0.59, 1.09]	27.5%	0.89 [0.71, 1.13]
Middle	2.0%	1.45 [0.64, 3.31]	12.0%	0.75 [0.54, 1.04]	23.4%	0.72 [0.56, 0.92]
Middle low	1.8%	1.27 [0.52, 3.09]	10.1%	0.62 [0.43, 0.89]	21.6%	0.65 [0.49, 0.85]
Low (bottom quintile)	3.2%	2.31 [0.99, 5.38]	9.5%	0.58 [0.38, 0.89]	21.5%	0.65 [0.47, 0.88]
		p=0.21		p=0.03		p=0.002

Reference categories for outcomes: nicotine dependence ‘non-smoker/low/very low dependence’; alcohol consumption ‘low risk/hazardous’;other illicit drug use ‘never used/not used in last 3 months’

Table S5. Early tobacco / alcohol use and risk of 21-year outcomes

	Nicotine dependence		Harmful alcohol consumption		Other illicit drug use	
	% with outcome	OR [95% CI]	% with outcome	OR [95% CI]	% with outcome	OR [95% CI]
Self-report smoking at 12yr 10mn						
No	2.0%	1.00 ref.	11.9%	1.00 ref.	11.4%	1.00 ref.
Yes	7.7%	4.16 [2.37, 7.28]	22.9%	2.20 [1.56, 3.09]	24.3%	2.49 [1.73, 3.59]
		p < 0.001		p < 0.001		p < 0.001
Self-report alcohol use at 12yr 10mn						
None	1.9%	1.00 ref.	11.2%	1.00 ref.	10.0%	1.00 ref.
Less than weekly	2.6%	1.43 [0.67, 3.07]	17.8%	1.71 [1.23, 2.37]	19.2%	2.25 [1.61, 3.15]
Weekly +	9.3%	5.41 [3.16, 9.25]	19.6%	1.93 [1.35, 2.78]	24.0%	2.86 [1.97, 4.15]
		p < 0.001		p < 0.001		p < 0.001

Reference categories for outcomes: nicotine dependence ‘non-smoker/low/very low dependence’; alcohol consumption ‘low risk/hazardous’; other illicit drug use ‘never used/not used in last 3 months’
